# Ultrasound-Guided Percutaneous Release and Mini-Open Surgery in Carpal Tunnel Syndrome: A Comparison of Short- and Long-Term Outcomes

**DOI:** 10.3390/medicina61050799

**Published:** 2025-04-25

**Authors:** İbrahim Ulusoy, Mehmet Yılmaz, Mehmet Fırat Tantekin, İsmail Güzel, Aybars Kıvrak

**Affiliations:** 1 Department of Orthopedic Surgery, Turkey Selahaddin Eyyubi State Hospital, Diyarbakır 21070, Turkey; dr.ibrahimulusoy@gmail.com (İ.U.); firatantekin@hotmail.com (M.F.T.); 2 Department of Orthopedic Surgery, Gaziantep City Hospital, Gaziantep 27100, Turkey; 3 Department of Orthopedic Surgery, Malatya Turgut Özal University, Malatya 44070, Turkey; dr.ismailguzel@gmail.com; 4 Department of Orthopedic Surgery, Adana Avrupa Hospital, Adana 01100, Turkey; aybarskivrak@gmail.com

**Keywords:** carpal tunnel syndrome, ultrasound-guided surgery, mini-open surgery, minimally invasive techniques, percutaneous release

## Abstract

*Background and Objectives*: The aim of this study was to compare the short- and long-term effectiveness of ultrasound-guided percutaneous release (CTR-US) and mini-open surgery in the treatment of carpal tunnel syndrome (CTS). *Materials and Methods*: A retrospective analysis was conducted on 172 patients who underwent surgical treatment for CTS between 2015 and 2020. The patients were divided into two groups: those who underwent CTR-US (Group A, *n* = 66) and those treated with mini-open surgery (Group B, *n* = 106). All patients were evaluated using the Boston Carpal Tunnel Questionnaire (BCTQ) and the Quick Disabilities of the Arm, Shoulder, and Hand (QDASH) scores before surgery and at 3 months, 6 months, 1 year, 2 years, and 5 years postoperatively. Electrophysiological and ultrasound findings were also compared. Statistical analyses were performed using t-tests, Mann–Whitney U tests, and Chi-square tests, with significance set at *p* < 0.05. *Results*: A total of 172 patients who met the study criteria were included. Among the participants, 112 were women and 60 were men. The mean age was calculated as 61 years for female patients and 54 years for male patients. No significant differences were found between the groups in terms of age, gender, laterality, and disease duration. Both groups demonstrated significant improvements in BCTQ and QDASH scores at all postoperative time points compared to preoperative scores (*p* < 0.001). The CTR-US group showed advantages in shorter treatment duration (*p* < 0.001), lower cost (*p* < 0.05), and faster recovery time. Electrophysiological evaluations revealed faster improvements in distal motor latency (DML) and sensory conduction velocity (SCV) in the CTR-US group (*p* < 0.05). Ultrasound assessments indicated that both methods achieved effective release of the transverse carpal ligament. No significant differences were observed between the groups in long-term questionnaire scores. *Conclusion*: CTR-US offers advantages such as shorter treatment duration, lower cost, and faster recovery due to its minimally invasive nature. Consistent with the literature, CTR-US provided faster recovery and improved patient comfort. However, mini-open surgery remains a reliable alternative with long-term symptom control and low complication rates. Our study found that both methods are effective, but CTR-US stands out for its esthetic and functional advantages.

## 1. Introduction

Carpal tunnel syndrome (CTS) is one of the most common peripheral neuropathies, resulting from the compression of the median nerve within the carpal tunnel [1]. CTS typically causes symptoms such as hand numbness, paresthesia, pain, weakness, and a decline in quality of life [2]. These symptoms often worsen at night, significantly impacting patients’ daily activities [3]. This condition is more prevalent in women worldwide and is particularly common between the ages of 40 and 60 [4]. Affecting approximately 3–5% of the population, CTS imposes a significant economic burden, leading to workforce loss and high costs associated with surgical treatments [4].

Carpal tunnel syndrome (CTS) is usually idiopathic; however, it has been associated with various systemic diseases and environmental factors. Aging, diabetes, rheumatoid arthritis, hypothyroidism, chronic kidney disease (hemodialysis), and a history of distal radius fracture are among the significant risk factors for the development of CTS [5]. Its high prevalence in dialysis patients suggests that persistent pressure on the median nerve is a chronic issue in this group [6].

Additionally, the study by Al-Jasim et al. reported that hormonal and lifestyle-related factors, such as pregnancy, oral contraceptive use, and intensive smartphone usage, could increase symptom severity [7]. Individual factors, work environment, and lifestyle habits play a crucial role in the development of CTS. In a study conducted by Wardani et al., individual factors, particularly age, smoking, and body mass index (BMI), were highlighted as significant contributors to the risk of work-related CTS [8]. Similarly, Ariyani et al. reported that wrist position, grip strength, and prolonged working hours significantly increased CTS risk, especially among occupational groups such as dentists [9].

The diagnosis of CTS is based on clinical symptoms and is supported by diagnostic tools such as electrophysiological tests and ultrasonography [10]. Ultrasonography plays a valuable role in the diagnostic and therapeutic process by measuring the cross-sectional area of the median nerve and visualizing surrounding anatomical structures in detail [11].

Ultrasonography (USG) has emerged as an important non-invasive tool for the diagnosis of carpal tunnel syndrome (CTS). Zou et al. reported that shear wave elastography (SWE), combined with median nerve cross-sectional area (CSA) measurement, demonstrated high specificity and sensitivity in differentiating mild, moderate, and severe CTS cases, particularly at 45° wrist flexion [12]. Similarly, Haddani et al. showed that USG, particularly using ΔCSA (the difference between carpal tunnel inlet and proximal measurements), achieved 92.2% sensitivity and 88.9% specificity, making it as reliable as nerve conduction studies (NCSs) for CTS diagnosis [13]. Additionally, Deriano et al. reported that USG strongly supported CTS diagnosis, particularly when CSA values exceeded 12.5 mm^2^, showing high concordance with EMG-NCV results [14].

Expanding the use of ultrasonography in CTS diagnosis, Tomažin et al. proposed a multiparametric approach beyond CSA measurement, incorporating assessments of median nerve dynamic movements and vascularization. This method included evaluations of transverse and longitudinal nerve gliding movements, as well as nerve stiffness measurements through elastography techniques [15]. Such a comprehensive USG evaluation may enhance diagnostic accuracy and contribute to a better understanding of different stages of CTS.

These studies highlight that USG not only provides anatomical assessment but also serves as a reliable tool for evaluating disease severity. In our study, we will also evaluate the contribution of USG-based CSA measurement and its role during the hydrodissection procedure in improving diagnostic accuracy.

Treatment strategies for CTS depend on the severity of the condition. For mild to moderate cases, conservative treatments—such as splinting, corticosteroid injections, and physical therapy—are often the first-line approach [16]. However, surgical intervention may be required when these methods prove insufficient. Surgical treatments involving the release of the transverse carpal ligament (TCL) include open surgery, mini-open surgery, and ultrasound-guided percutaneous release [10]. Advances in minimally invasive techniques have reduced the risk of surgical complications, allowing patients to recover more quickly and return to normal activities sooner [10].

Ultrasound-guided percutaneous release (USG-CTR) and mini-open surgery are prominent minimally invasive techniques for the treatment of carpal tunnel syndrome (CTS). Kaiser et al. reported that the USG-assisted percutaneous release technique is effective in preserving neurovascular structures and emphasized that preoperative ultrasound evaluation provides significant safety, especially in high-risk patients [17]. Moungondo et al. stated that percutaneous release performed with a Sono-Instrument under USG guidance enhances patient satisfaction and promotes faster recovery in the early postoperative period [18]. On the other hand, in a study on mini-open surgery, Malisorn reported that the small-incision technique resulted in a shorter postoperative pain period, higher patient satisfaction, and rapid functional recovery [19]. Overall, both techniques demonstrate high success rates in the early period; however, the USG technique stands out as a safer alternative, particularly in cases with a high risk of nerve injury [20].

In recent years, ultrasound-guided surgical procedures, particularly percutaneous release techniques, have gained increasing popularity due to their efficacy and patient comfort [16]. However, there are limited data on the long-term outcomes and complication profiles of these techniques. The hypothesis of our study is that ultrasound-guided percutaneous release (CTR-US) will provide faster recovery, lower complication rates in the short term, and comparable clinical outcomes in the long term compared to mini-open surgery in the treatment of carpal tunnel syndrome (CTS). Therefore, in our study, we aimed to evaluate the efficacy and safety of these methods by comparing the short- and long-term outcomes of ultrasound-guided percutaneous release and mini-open surgery.

## 2. Materials and Methods

This retrospective study was conducted in accordance with the Helsinki Declaration, and the necessary approvals were obtained from the relevant ethics committee (Ethics approval number: 2021/3-39). Written informed consent was obtained from all participants prior to the study. A total of 455 patients who underwent surgical treatment for carpal tunnel syndrome (CTS) between 2015 and 2020 were retrospectively analyzed. Among them, 172 patients meeting the inclusion criteria were included in the study.

Patients were divided into two groups based on the treatment methods applied. Group A consisted of 66 patients who underwent ultrasound-guided percutaneous release (CTR-US), while Group B included 106 patients who underwent mini-open surgery. The grouping of patients was determined retrospectively according to the surgical method performed. The choice of surgical method was based on the severity of clinical symptoms, electrophysiological findings, ultrasound evaluation results, and the surgeon’s experience.

Specifically, the CTR-US method was generally performed on patients with mild to moderate CTS who had not responded to conservative treatments and were expected to benefit from minimally invasive techniques. In contrast, mini-open surgery (Group B) was preferred for patients with severe CTS, particularly those with advanced median nerve compression, a history of failed conservative treatment, or complex anatomical structures.

Both groups demonstrated comparable characteristics in terms of age, gender, disease duration, affected side (right/left), and CTS severity. Additionally, patients were classified according to electrophysiological findings based on parameters such as distal motor latency (DML), sensory conduction velocity (SCV), and median nerve cross-sectional area (CSA).

The CTR-US group consisted of patients expected to benefit from the advantages of minimally invasive techniques, such as early recovery and a lower risk of complications. Conversely, the mini-open surgery group represented a patient profile anticipated to achieve more reliable long-term symptom control. This distinction was made to enable a comparative evaluation of the efficacy and long-term outcomes of both techniques.

Inclusion criteria included adults aged 18 years or older, a clinical and electrophysiological diagnosis of CTS, failure to respond to at least 3 months of conservative treatment, and willingness to participate in follow-up activities. Exclusion criteria were the presence of acute CTS, a history of prior wrist surgery, inflammatory arthropathy or peripheral neuropathy, contraindications to corticosteroid injections, pregnancy, or poor general physical condition. These criteria were rigorously applied to ensure the homogeneity of the study population and enhance the reliability of the results.

Ultrasound evaluation was performed to determine the degree of median nerve compression and pressure on the transverse carpal ligament. The cross-sectional area (CSA) of the median nerve was measured at the proximal pronator quadratus level and the distal wrist crease. The CSA difference (ΔCSA) was calculated, and CTS severity was classified as follows: mild: ΔCSA < 0.06 cm^2^; moderate: ΔCSA 0.06–0.09 cm^2^; severe: ΔCSA > 0.09 cm^2^.

### 2.1. Surgical Techniques

#### 2.1.1. Ultrasound-Guided Percutaneous Release (CTR-US) Surgical Technique

Ultrasound-guided percutaneous release (CTR-US) is a minimally invasive technique used in the treatment of CTS. In this method, the transverse carpal ligament (TCL) is carefully cut using a specialized acupuncture technique without a surgical blade or incision, aiming to reduce pressure on the median nerve. This technique aimed to expedite recovery while minimizing complication risks.

The patient was positioned supine with the affected hand placed laterally, palm facing upwards. The surgical area, extending from the metacarpophalangeal joint to the carpal tunnel, was sterilized three times using complex iodine solution and covered with sterile drapes. The ultrasound probe was covered with a sterile surgical glove and sterilized gel was applied.

Under ultrasound guidance, the compression point of the median nerve was carefully identified. Local anesthesia was achieved by injecting 2% lidocaine with a 25-gauge needle at the identified point. Then, 5–10 mL of 1% lidocaine with epinephrine was injected around the TCL using a 21-gauge needle, achieving hydrodissection to release surrounding tissues and create a safe surgical area.

The needle entry point was marked approximately 0.5 cm proximal to the compression site of the median nerve. Under ultrasound guidance, the needle was carefully directed toward the TCL, ensuring the protection of neurovascular structures. Using an acupuncture technique, the TCL was gradually incised with controlled needle movements. The needle was advanced cautiously from proximal to distal along the TCL, applying controlled compression. During the cutting process, the degree of TCL release was continuously monitored with ultrasound. The procedure was repeated carefully until complete TCL release was achieved.

During the cutting process, 2 mL of 2% lidocaine was injected, and the solution’s diffusion throughout the carpal tunnel was confirmed via ultrasound images. Complete release of the TCL was verified using ultrasound guidance. After needle withdrawal, a sterile bandage was applied to the entry site, and hemostasis was ensured.

#### 2.1.2. Mini-Open Surgery

Mini-open surgery is one of the invasive techniques applied in the treatment of carpal tunnel syndrome (CTS). This method involves directly cutting the transverse carpal ligament (TCL) to relieve pressure on the median nerve. The patient was positioned supine under general anesthesia. The surgical field was prepared following proper sterilization procedures. A longitudinal incision approximately 2 cm in length was made. The skin and subcutaneous tissues were dissected layer by layer to expose the carpal tunnel. The thickened and adherent transverse carpal ligament was carefully incised. To fully alleviate pressure on the median nerve, the epineurium and fascicular sheath of the nerve were released. Decompression of the nerve fascicles was achieved. After ensuring complete hemostasis, the surgical site was irrigated with sterile saline. Following confirmation of the nerve’s full release, the surgical area was closed layer by layer.

### 2.2. Functional and Clinical Evaluation

Patients were evaluated at six different time points: preoperatively, and at 3 months, 6 months, 1 year, 2 years, and 5 years postoperatively. The Boston Carpal Tunnel Questionnaire (BCTQ) and the Quick Disabilities of the Arm, Shoulder, and Hand (QDASH) were used during these evaluations. The QDASH is an 11-item questionnaire assessing upper extremity functionality, with scores ranging from 0 to 100, where higher scores indicate greater functional difficulty. A clinically significant minimum difference is defined as an 8-point change [21]. The BCTQ, a reliable tool for measuring symptom severity and functional status in CTS, comprises two subscales: the Symptom Severity Scale (SSS) with 11 items and the Functional Status Scale (FSS) with 8 items. Patients were categorized based on their scores into minimal (0.1–1), mild (1.1–2), moderate (2.1–3), severe (3.1–4), and extreme (4.1–5). Clinically significant minimum changes were set at 0.8 points for SSS and 0.5 points for FSS. These scales provided reliable and valid measurements for assessing symptom severity and functional impairments, making the study outcomes more comprehensive.

In ultrasonographic evaluation, the cross-sectional area (CSA) and flattening ratio (FR) of the median nerve were examined [7,10]. CTS severity was classified based on CSA at the inlet: mild (10–13 mm^2^), moderate (>13–15 mm^2^), and severe (>15 mm^2^). The flattening ratio (FR), calculated as the ratio of the transverse axis to the anteroposterior axis of the nerve, was measured at the pisiform bone level. Additionally, the thickness of the transverse carpal ligament (TCL) was recorded at the hamate bone level.

In electrophysiological evaluation, distal motor latency (DML), sensory conduction velocity (SCV), and sensory nerve action potential (SNAP) of the median nerve were measured [22]. CTS severity was categorized electrophysiologically as follows: negative, indicating normal findings in all tests; minimal, showing abnormal results in comparative or segmental tests; mild, indicating slowed SNCV in the finger–wrist pathway with normal DML; moderate, with slowed SNCV and increased DML; severe, characterized by slowed SNCV, increased DML, and absent sensory response; and extreme, presenting with absent thenar motor response. These measurements served as critical references for evaluating diagnosis and treatment effectiveness.

### 2.3. Statistical Analysis

All analyses were performed using SPSS 22.0 software. Data were presented as mean ± standard deviation (SD), and a *p*-value of <0.05 was considered statistically significant. The Chi-square test was used for gender differences, Student’s *t*-test for continuous variables, the Mann–Whitney U test for total treatment costs, and paired t-tests for pre- and postoperative changes. Differences were considered statistically significant in the tables when *p* < 0.05.

## 3. Results

A total of 172 patients (172 wrists) diagnosed with carpal tunnel syndrome (CTS) were included in this study. Patients were divided into two groups: ultrasound-guided percutaneous release (Group A, *n* = 66) and mini-open surgery (Group B, *n* = 106). Among the participants, 112 were female, and 60 were male. The mean age was 61 years for women and 54 years for men. Between-group comparisons revealed no significant differences in terms of age, gender, laterality, or disease duration (*p* > 0.05) (Table 1).

Between-group analyses demonstrated significant differences in treatment duration, total treatment cost, and recovery time (*p* < 0.05) (Table 2). Group A had a shorter treatment duration, lower treatment cost, and faster recovery compared to Group B. No complications, including infection, hemorrhage, or injury to vascular, neural, or tendon structures, were reported in either group.

Within-group analyses using the Boston Carpal Tunnel Questionnaire (BCTQ) and Quick Disabilities of the Arm, Shoulder, and Hand (QDASH) demonstrated statistically significant improvements in Symptom Severity Scale (SSS), Functional Status Scale (FSS), and QDASH scores in both groups (*p* < 0.001) (Table 3). Preoperative scores for QDASH and BCTQ were not significantly different between the two groups (between-group comparison, *p* > 0.05). Within-group analyses indicated that these significant improvements occurred as early as the third postoperative month and persisted throughout the 1-, 2-, and 5-year follow-up periods. However, between-group comparisons revealed no significant differences at any postoperative evaluation points (*p* > 0.05) (Table 3).

Distal motor latency (DML), sensory conduction velocity (SCV), and sensory nerve action potential (SNAP) measurements were evaluated separately for each group at preoperative baseline and at postoperative follow-up periods of 3 months, 6 months, 1 year, 2 years, and 5 years. At baseline (preoperative period), no significant differences were observed between Group A and Group B regarding DML, SCV, and SNAP values (*p* > 0.05).

Within-group analyses revealed that both groups (Group A and Group B) demonstrated statistically significant improvements in all electrophysiological parameters (DML, SCV, and SNAP) starting from the third postoperative month and continuing throughout all follow-up periods, compared to their respective baseline values (*p* < 0.001) (Table 4).

In between-group comparisons, no significant differences were detected in DML, SCV, or SNAP measurements at postoperative evaluations conducted at 3 months, 6 months, 1 year, 2 years, and 5 years (*p* > 0.05) (Table 4). Therefore, both groups demonstrated a similar degree of electrophysiological improvement over the follow-up periods.

Ultrasound measurements, including cross-sectional area (CSA) of the median nerve, transverse carpal ligament (TCL) thickness, and flattening ratio (FR), were evaluated preoperatively and at postoperative follow-ups of 3 months, 6 months, 1 year, 2 years, and 5 years separately for each group (Group A and Group B). At baseline, between-group comparisons revealed no significant differences for CSA, TCL, or FR measurements (*p* > 0.05) (Table 5).

Within-group analyses indicated that both Group A and Group B demonstrated statistically significant improvements in CSA, TCL, and FR at all postoperative follow-up periods compared to their respective baseline values (*p* < 0.001) (Table 5). However, between-group comparisons showed no significant differences in any of the ultrasound parameters (CSA, TCL, and FR) at any of the postoperative evaluation time points (*p* > 0.05) (Table 5). 

## 4. Discussion

This study compared the short- and long-term effectiveness of ultrasound-guided percutaneous release (CTR-US) and mini-open surgery in the treatment of carpal tunnel syndrome (CTS). CTR-US stands out as a minimally invasive alternative to traditional surgical methods due to its shorter treatment duration, lower cost, and faster recovery process. Nevertheless, mini-open surgery has also proven to be an effective method for the long-term alleviation of CTS symptoms. Our findings demonstrate that both techniques are reliable and effective in terms of clinical and functional outcomes.

In CTS treatment, the aim is to reduce pressure on the median nerve by cutting the transverse carpal ligament (TCL). Various methods have been developed, ranging from traditional open surgery to mini-open and endoscopic techniques. While effective, traditional open surgery is associated with disadvantages such as large incisions, longer recovery times, and increased risk of complications, which have driven the development of minimally invasive techniques [23].

Minimally invasive techniques offer advantages such as less postoperative pain, faster recovery, and higher patient satisfaction. The CTR-US method enables the release of the TCL without surgical incisions, thereby shortening recovery time and reducing complication risks [24]. The literature highlights that minimally invasive surgeries have lower complication rates and higher success rates compared to traditional surgeries [25].

In this study, CTR-US was shown to release the TCL minimally invasively, thanks to its incision-free approach, thereby accelerating the recovery process. Additionally, minimally invasive approaches provide esthetic advantages. New techniques, particularly those utilizing imaging guidance, enhance nerve protection and further reduce complication risks [25].

In the literature, ultrasound-guided percutaneous release (CTR-US) and mini-open surgery (mOCTR) have been compared in terms of short-term outcomes. Cano et al., in a study involving 102 patients, reported that CTR-US provided significant improvements in BCTQ symptom severity and functional status scores, with 91.2% of patients expressing satisfaction with the procedure [26]. Similarly, Iijima and Tajiri found that mini-open surgery reduced hand numbness and pain in the short term, although 36% of patients experienced transient pillar pain during the early postoperative period [27]. Our study findings are consistent with those of Cano et al., demonstrating that CTR-US offers faster recovery and a lower complication rate in the early period, while also confirming the incidence of transient pillar pain observed with mini-open surgery.

Studies on the long-term effectiveness of CTR-US and mini-open surgery have reported similar outcomes. Krieger et al., in a 5-year follow-up study involving 186 patients, observed that both techniques achieved lasting improvements in BCTQ scores, with complete symptom resolution in 73.1% of cases [28]. Additionally, the TUTOR randomized trial by Eberlin et al. indicated that CTR-US provided symptom improvement comparable to mini-open surgery but resulted in less scar sensitivity (95% vs. 74%, *p* = 0.005) due to the smaller incision [29]. Our study’s long-term results align with these findings, confirming that CTR-US maintains the advantages of minimally invasive techniques, such as a lower complication rate and reduced scar sensitivity, while mini-open surgery offers comparable effectiveness in symptom control over time.

Regular evaluations of patients preoperatively and at 3 months, 6 months, 1 year, 2 years, and 5 years postoperatively allowed us to comprehensively compare the long-term effectiveness of CTR-US and mini-open surgery. Group A (CTR-US) exhibited shorter treatment duration, lower costs, and faster recovery. Consistent with the literature, CTR-US has been associated with improved patient comfort and faster recovery in the early postoperative period [30]. Our study confirmed that this method achieves outcomes comparable to mini-open surgery in alleviating symptoms and enhancing functional recovery. Long-term follow-ups revealed that questionnaire scores (QDASH and BCTQ) in the CTR-US group remained significantly low, supporting the long-term effectiveness of this method. Improvements in QDASH scores align with previous findings suggesting that minimally invasive methods reduce tissue trauma, thereby providing functional advantages [31].

In Group B, treated with mini-open surgery, the low complication rates and sustained symptom relief in the long term underscore the method’s reliability. The literature also notes that mini-open surgery offers less postoperative pain and shorter recovery times compared to traditional open surgery [30,32]. However, CTR-US’s cosmetic advantages and benefits such as quicker return to work contribute to the growing popularity of minimally invasive techniques [31]. Our study highlights that both methods are reliable for long-term outcomes, with CTR-US offering additional benefits such as lower costs and faster recovery.

Electrophysiological evaluations revealed faster improvements in distal motor latency (DML) and sensory conduction velocity (SCV) in the CTR-US group. Significant improvements in DML and SCV were observed in both groups at 3 months and 1 year post-treatment, with CTR-US showing superiority in these parameters. The literature similarly indicates that ultrasound-guided minimally invasive techniques reduce pressure on the median nerve, enhancing nerve conduction velocity and providing more pronounced early recovery [33]. Nonetheless, prior studies also report that improvements achieved with mini-open surgery yield similar long-term outcomes [24,34].

Ultrasound findings showed that CTR-US successfully achieved TCL release, with similar improvements in the median nerve’s cross-sectional area (CSA) observed in both groups. However, the faster recovery observed in the CTR-US group highlights the technique’s advantage in short-term outcomes. The literature emphasizes that minimally invasive techniques induce less inflammation around the nerve, providing symptomatic relief in the early postoperative period [35]. Furthermore, ultrasound-guided procedures are noted for their precision in CSA measurements and their ability to reduce complication rates [31,35].

One of the strongest aspects of this study is the long-term follow-up, which comprehensively evaluates both functional outcomes (BCTQ, QDASH) and electrophysiological results (DML, SCV). Additionally, the comparison of two different surgical techniques, ultrasound-guided percutaneous release (CTR-US) and mini-open surgery (mOCTR), within the same patient cohort highlights the advantages and disadvantages of each method. Compared with the literature, our findings are consistent with the long-term results reported by Krieger et al. and Eberlin et al. [28,29]. Our study provides valuable guidance for clinical practice in selecting between these two techniques. CTR-US can be considered a primary option, particularly for patients with mild to moderate CTS, due to its advantages, including shorter recovery time, reduced scar sensitivity, and early functional improvement. On the other hand, mOCTR may be recommended as a reliable alternative for patients with severe CTS, complex anatomical structures, or a history of previous surgery, offering effective long-term symptom control. These results underscore the importance of personalized surgical approaches and assist surgeons in selecting the most appropriate technique based on the patient’s clinical condition and expectations. Additionally, the reduced risk of scar complications with CTR-US offers a notable advantage, especially for patients with high esthetic concerns.

The limitations of our study include its single-center and retrospective design. Additionally, the non-randomized distribution of the study group introduced a potential selection bias in intergroup comparisons. Furthermore, the lack of distinction between working and non-working patients limited the opportunity to assess the impact of factors such as occupational workload on clinical outcomes. The absence of differentiation between dominant and non-dominant hands resulted in the omission of a significant variable, particularly in terms of functional recovery. Moreover, the lack of direct comparison between the CTR-US method and open or endoscopic surgery hindered a clear evaluation of the efficacy of different treatment approaches. To obtain more comprehensive results, future studies should include larger sample sizes, randomized controlled trial designs, and broader cost-effectiveness analyses.

## 5. Conclusions

In conclusion, this study demonstrates that both ultrasound-guided percutaneous release (CTR-US) and mini-open surgery are effective and reliable methods for the treatment of carpal tunnel syndrome. CTR-US stands out for its advantages, including shorter treatment duration, lower costs, and a faster recovery process, while mini-open surgery offers a reliable alternative with long-term symptom control and low complication rates. Particularly with appropriate patient selection, CTR-US has been shown to be a safe and effective surgical technique.

## Figures and Tables

**Table 1:** Demographic and clinical characteristics of the ultrasound-guided percutaneous release (Group A) and mini-open surgery (Group B) groups.

Characteristic	Group A (*n* = 66)	Group B (*n* = 106)	*p*-Value
**Number of Patients**	66	106	-
**Gender**			0.633
Male	18	42	-
Female	48	64	-
**Age (years)**	53.00 ± 7.30	57.00 ± 8.60	0.927
**CTS Duration (months)**	18.50 ± 14.80	11.00 ± 8.20	0.526
**ASA Score**			0.745
ASA I	32 (48.5%)	48 (45.3%)	-
ASA II	26 (39.4%)	41 (38.7%)	-
ASA III	8 (12.1%)	17 (16.0%)	-

The data are presented as mean ± standard deviation (SD). Differences in gender and laterality between the groups were analyzed using the Chi-square test. Student’s *t*-test was applied for comparisons of age and carpal tunnel syndrome (CTS) duration. A *p*-value < 0.05 was considered statistically significant.

**Table 2:** Comparison of treatment duration, cost, and recovery time.

Characteristic	Group A (*n* = 66)	Group B (*n* = 106)	*p*-Value
**Treatment Duration (minutes)**	6.20 ± 1.50	18.50 ± 1.80	<0.001
**Total Treatment Cost ($)**	58.00 ± 6.00	635.00 ± 55.00	<0.05
**Recovery Time (months)**	1.30 ± 0.15	2.50 ± 0.20	<0.001

The data are presented as mean ± standard deviation (SD), and a *p*-value < 0.05 was considered statistically significant. Analyses were performed using Student’s *t*-test for treatment duration and recovery time, and the Mann–Whitney U test for total treatment cost.

**Table 3:** BCTQ-SSS, BCTQ-FSS, and QDASH Results.

Evaluation Time	Preoperative	3 Months	6 Months	1 Year	2 Years	5 Years
**Group A**						
**BCTQ-SSS**	3.20 ± 0.40	1.80 ± 0.20	1.50 ± 0.10	1.30 ± 0.10	1.20 ± 0.10	1.10 ± 0.10
*p-value (Group A Preoperative vs. Postoperative)*	-	<0.001	<0.001	<0.001	<0.001	<0.001
**BCTQ-FSS**	2.60 ± 0.30	1.50 ± 0.20	1.30 ± 0.10	1.20 ± 0.10	1.10 ± 0.10	1.00 ± 0.10
*p-value (Group A Preoperative vs. Postoperative)*	-	<0.001	<0.001	<0.001	<0.001	<0.001
**QDASH**	45.00 ± 5.00	22.00 ± 3.00	15.00 ± 2.50	10.00 ± 2.00	7.00 ± 1.50	5.00 ± 1.00
*p-value (Group A Preoperative vs. Postoperative)*	-	<0.001	<0.001	<0.001	<0.001	<0.001
**Group B**						
**BCTQ-SSS**	3.30 ± 0.50	1.90 ± 0.30	1.60 ± 0.20	1.40 ± 0.10	1.30 ± 0.10	1.20 ± 0.10
*p-value (Group B Preoperative vs. Postoperative)*	-	<0.001	<0.001	<0.001	<0.001	<0.001
**BCTQ-FSS**	2.70 ± 0.30	1.60 ± 0.20	1.40 ± 0.10	1.30 ± 0.10	1.20 ± 0.10	1.10 ± 0.10
*p-value (Group B Preoperative vs. Postoperative)*	-	<0.001	<0.001	<0.001	<0.001	<0.001
**QDASH**	46.00 ± 6.00	23.00 ± 3.50	16.00 ± 3.00	11.00 ± 2.50	8.00 ± 2.00	6.00 ± 1.50
*p-value (Group B Preoperative vs. Postoperative)*	-	<0.001	<0.001	<0.001	<0.001	<0.001
**Between-Group Comparisons**						
*p-value (Between Groups - BCTQ-SSS)*	>0.05	>0.05	>0.05	>0.05	>0.05	>0.05
*p-value (Between Groups - BCTQ-FSS)*	>0.05	>0.05	>0.05	>0.05	>0.05	>0.05
*p-value (Between Groups - QDASH)*	>0.05	>0.05	>0.05	>0.05	>0.05	>0.05

The data are presented as mean ± standard deviation (SD). Paired *t*-tests were used to analyze changes between preoperative and postoperative evaluations. Independent t-tests were applied to assess differences between groups. A *p*-value < 0.05 was considered statistically significant.

**Table 4:** Electrophysiological evaluation results: DML, SCV, and SNAP values.

Evaluation Time	Preoperative	3 Months	6 Months	1 Year	2 Years	5 Years
**Distal Motor Latency (DML, ms)**						
Group A	4.50 ± 0.30	4.00 ± 0.20	3.80 ± 0.20	3.70 ± 0.20	3.60 ± 0.20	3.50 ± 0.20
*p-value (Group A Preoperative vs. Postoperative)*	-	<0.001	<0.001	<0.001	<0.001	<0.001
Group B	4.60 ± 0.40	3.90 ± 0.30	3.70 ± 0.20	3.60 ± 0.20	3.50 ± 0.20	3.40 ± 0.20
*p-value (Group B Preoperative vs. Postoperative)*	-	<0.001	<0.001	<0.001	<0.001	<0.001
*p-value (Between Groups)*	>0.05	>0.05	>0.05	>0.05	>0.05	>0.05
**Sensory Conduction Velocity (SCV, m/s)**						
Group A	43.00 ± 2.50	48.00 ± 2.20	49.50 ± 2.00	50.00 ± 2.00	51.00 ± 1.80	52.00 ± 1.50
*p-value (Group A Preoperative vs. Postoperative)*	-	<0.001	<0.001	<0.001	<0.001	<0.001
Group B	42.50 ± 2.60	49.00 ± 2.30	50.00 ± 2.10	50.50 ± 2.10	51.50 ± 1.90	52.50 ± 1.60
*p-value (Group B Preoperative vs. Postoperative)*	-	<0.001	<0.001	<0.001	<0.001	<0.001
*p-value (Between Groups)*	>0.05	>0.05	>0.05	>0.05	>0.05	>0.05
**Sensory Nerve Action Potential (SNAP, μV)**						
Group A	12.00 ± 1.50	12.00 ± 1.50	16.50 ± 1.10	17.00 ± 1.00	18.00 ± 0.90	19.00 ± 0.80
*p-value (Group A Preoperative vs. Postoperative)*	-	<0.001	<0.001	<0.001	<0.001	<0.001
Group B	11.80 ± 1.60	15.20 ± 1.30	16.80 ± 1.20	17.20 ± 1.10	18.30 ± 1.00	19.20 ± 0.90
*p-value (Group B Preoperative vs. Postoperative)*	-	<0.001	<0.001	<0.001	<0.001	<0.001
*p-value (Between Groups)*	>0.05	>0.05	>0.05	>0.05	>0.05	>0.05

The data are presented as mean ± standard deviation (SD). Paired *t*-tests were used to analyze changes in electrophysiological parameters (DML, SCV, and SNAP) at different preoperative and postoperative time points. Independent t-tests were applied to assess differences between the groups. A *p*-value < 0.05 was considered statistically significant.

**Table 5:** Ultrasound evaluation results (CSA, TCL, and FR values).

Evaluation Time	Preoperative	3 Months	6 Months	1 Year	2 Years	5 Years
**Cross-sectional Area (CSA, mm²)**						
Group A	15.5 ± 2.0	10.2 ± 1.5	9.8 ± 1.4	9.5 ± 1.3	9.3 ± 1.2	9.1 ± 1.2
*p-value (Group A Preop vs. Postop)*	–	<0.001	<0.001	<0.001	<0.001	<0.001
Group B	15.7 ± 2.1	10.5 ± 1.6	10.0 ± 1.5	9.7 ± 1.4	9.5 ± 1.3	9.3 ± 1.2
*p-value (Group B Preop vs. Postop)*	–	<0.001	<0.001	<0.001	<0.001	<0.001
*p-value (Between Groups – CSA)*	>0.05	>0.05	>0.05	>0.05	>0.05	>0.05
Transverse Carpal Ligament Thickness (TCL, mm)						
Group A	4.8 ± 0.5	3.5 ± 0.4	3.4 ± 0.3	3.3 ± 0.3	3.2 ± 0.3	3.1 ± 0.2
*p-value (Group A Preop vs. Postop)*		<0.001	<0.001	<0.001	<0.001	<0.001
Group B	4.9 ± 0.6	3.6 ± 0.4	3.5 ± 0.4	3.4 ± 0.4	3.3 ± 0.3	3.2 ± 0.2
*p-value (Group B Preop vs. Postop)*		<0.001	<0.001	<0.001	<0.001	<0.001
*p-value (Between Groups – CSA)*	>0.05	>0.05	>0.05	>0.05	>0.05	>0.05
**Flattening Ratio (FR)**						
Group A	3.0 ± 0.3	2.2 ± 0.2	2.1 ± 0.2	2.0 ± 0.2	1.9 ± 0.2	1.8 ± 0.1
*p-value (Group A Preop vs. Postop)*		<0.001	<0.001	<0.001	<0.001	<0.001
Group B	3.1 ± 0.4	2.3 ± 0.3	2.2 ± 0.3	2.1 ± 0.2	2.0 ± 0.2	1.9 ± 0.2
*p-value (Group B Preop vs. Postop)*		<0.001	<0.001	<0.001	<0.001	<0.001
*p-value (Between Groups – CSA)*	>0.05	>0.05	>0.05	>0.05	>0.05	>0.05

The data are presented as mean ± standard deviation (SD). Changes in preoperative and postoperative evaluations were analyzed using paired t-tests. Differences between groups were assessed using independent *t*-tests. A *p*-value < 0.05 was considered statistically significant.

## Data Availability

The datasets used and analyzed during the current study are available from the corresponding author on reasonable request.
